# The Mechanism of Downregulation of Twist1 Inhibiting Trophoblast Invasion and Aggravating the Development of Preeclampsia

**DOI:** 10.3389/fsurg.2022.862716

**Published:** 2022-03-17

**Authors:** Shuangjian Yang, Wenjuan Tong, Yi Li

**Affiliations:** The First Affiliated Hospital, Department of Gynecology and Obstetrics, Hengyang Medical School, University of South China, Hengyang, China

**Keywords:** preeclampsia, transcription factor Twist1, placental trophoblast cells, invasion ability, urine protein

## Abstract

To study the expression of under expressed transcription factor Twist1 in preeclampsia (PE) and its effect on the invasion of placental trophoblast cells and to explore its related mechanism on the development of PE by establishing a pregnant rat model. Methods: the villi were collected from the induced abortion in the first trimester (6–8 weeks), the normal placenta (18–20 weeks) induced by the second trimester, the term placenta tissue of normal pregnancy (37–40 weeks), and the placental tissue of patients with PE, to detect the expression of Twist1. Trophoblast cells were subjected to primary culture in placental tissues of normal pregnant women and placental tissues of PE patients. The invasion ability of the two groups of trophoblasts was detected, and the primary cultured trophoblasts were divided into two groups: an experimental group and a control group. Specific Twist1 siRNA was added to the experimental group, and no reagents were added to the control group. The above-mentioned cells were given different interventions. To explore the effect of Twist1 on trophoblast cell invasion, cells were cultivated for 72 h. The SD rats were conceived. After the pregnancy was stable, the SD rats in different groups were treated with different treatments (interference with Twist1), and the average systolic blood pressure and urine protein of the gestational mothers in the different treatment groups were measured at 1 week, 2 weeks, and full-term pregnancy. The expression of Twist1 in the placenta tissue of SD rats with different interventions at full-term pregnancy was detected. The results showed that Twist1 expression is down-regulated in PE, and the invasion ability of placental trophoblast cells in PE patients is weak. After inhibiting Twist1, the mean tail artery pressure and urine protein level of SD pregnant rats increase, showing a trend of PE. The mechanism may be related to the inhibition of the placenta by Twist1 Trophoblast cell invasion.

## Introduction

Preeclampsia (PE) is a special type of hypertensive disorder complicating pregnancy (HDCP). The main characteristics are hypertension, proteinuria, and edema after 20 weeks of pregnancy, and blood pressure returns to normal after delivery (1). Due to differences in geography, society, economy, ethnicity, and culture, the incidence is different in different regions. Worldwide, the incidence of PE is about 2–8%, while the incidence of PE in our country is relatively high, about 9.4%. Although more and more countries continue to improve the health of mothers and babies, more than 500,000 women die of pregnancy-related diseases every year, and about 10–15% of pregnant women die directly from PE and eclampsia (2–4).

The basic pathophysiological changes of PE are manifested as small vasospasm, vascular endothelial injury, reduced blood perfusion in the patient, multiple organs are damaged by ischemia and hypoxia, and insufficient placental perfusion can lead to fetal injury (5). It is currently believed that shallow placental implantation is the basis for the onset of PE, leading to long-term ischemia and oxidative stress in the placenta, releasing abnormal placental factors into the maternal blood circulation and inducing PE (6). Placental trophoblasts are the performers of the infiltration of the placenta into the endometrium and myometrium. In the first trimester, the placental trophoblast cells are in a physiological hypoxic state, and then the trophoblast cells gradually infiltrate the endometrium to the inner 1/3 of the muscle layer and enter the lumen of the uterine spiral arterioles to replace the vascular endothelium, resulting in enlargement of the lumen. The villi area of the placenta gradually expands, the blood supply resistance of the uterus–placenta decreases, the blood volume increases, and the hypoxic state of placental trophoblast cells is corrected. The increased apoptosis rate of placental trophoblast cells and decreased invasion ability will cause insufficient placental infiltration and uterine vascular remodeling, resulting in insufficient placental blood supply and oxidative stress in trophoblast cells, which ultimately leads to the occurrence of PE (7). Therefore, exploring the factors that regulate the activity and function of trophoblasts and their intervention methods are the key points to clarify the pathogenesis of PE and guide its prevention and treatment.

Abnormal trophoblast cell invasion and spiral artery remodeling disorder cause decreased placental perfusion, ischemia, and hypoxia, which is one of the most important pathogenesis of PE, and Twist1 can regulate cell differentiation and inhibit apoptosis in a variety of human tissues and tumors. Twist1 is widely expressed in placental trophoblasts, and its expression level shows a gestational week-dependent increase (8, 9). It is reported in the literature that Twist1 has the ability to promote the differentiation of trophoblasts (10), so we speculate that it may play an important role in the pathogenesis of placental-related disease-PE during pregnancy, but the correlation between the two needs to be further confirmed. Studies have shown (11) that Twist1 can directly regulate the activity and function of trophoblast cells, but the mechanism of action is unknown. Therefore, this study collected different placental tissues to detect the expression of Twist1 and explored the effect of Twist1 on the invasion of trophoblasts by isolating placental trophoblasts. It also analyzed the effect of Twist1 in SD-conceived rats and explored the relevant mechanism of Twist1's influence on PE. The specific report is as follows.

## Materials and Methods

### Clinical Samples

From July 2018 to July 2020, the experiment collected chorionic villi in the first trimester of pregnancy (6–8 weeks), normal placenta (18–20 weeks), normal pregnancy full-term placenta (37–40 weeks), and placental tissue of PE (34–37 weeks). PE is characterized by hypertension after 20 weeks of pregnancy, blood pressure ≥140/90 mmHg, accompanied by proteinuria (≥300 mg protein/24 h), liver and kidney damage, pulmonary edema, central nervous system abnormalities, or visual impairment. None of the subjects developed thrombocytopenia syndrome. The study excluded patients with smoking, drinking, chronic hypertension, nephritis, cardiovascular disease comorbidities, multiple pregnancies, fetal chromosomal abnormalities, or other fetal abnormalities. All cases were delivered by cesarean section before delivery. All cases signed an informed consent form for the use of placental specimens and the study was approved by the ethics committee of our hospital.

### Isolation and Culture of Placental Trophoblasts

The modified density gradient centrifugation method was used to separate and culture the trophoblast cells from the placental tissue of normal pregnancy and the placenta of patients with PE. The chorionic trophoblast tissue was cut and added in equal volumes of trypsin (2.5 g/L) and DNase (100 U/ml) in a 37°C water bath for 3 × 10 min and DMEM/F12 culture medium containing 10% fetal bovine serum was added to terminate the digested part and finally, the centrifugation was performed at 1,200 r/min for 5 min to obtain the cell suspension. The supernatant was discarded and resuspended in the DMEM/F12 medium culture. The cell suspension was divided into 2 parts, the first part was added to the cell Percoll separation solution, and the second part was added in the upper layer of the lymphocyte separation solution, and centrifuged at 1,500 r/min for 30 min with a centrifugal radius of 12 cm. A gray cloud-like cell layer at the junction of the different concentrations of the Percoll separation solution was observed, sucked the cell layer, and the cell layer between the lymphocyte separation solution and the culture solution was also observed. Both layers were washed two times with the culture solution and antibiotic saline and centrifuged at 1,200 r/min for 5 min (centrifugal radius = 12 cm). These cells were resuspended in the DMEM/F12 medium containing 20% fetal bovine serum, inoculated the culture plate at 1.0 × 105/ml, and continued to culture for 72 h in an incubator with 5% CO_2_, saturated humidity at 37°C.

### Identification of Trophoblasts

An inverted optical microscope (Jinan Jiawan Biotechnology Co., Ltd.) was used to observe the morphology of trophoblast cells; after cells were cultured for 72 h, 500 μl of freshly separated cell suspension was taken, fixed, and ruptured and 2 μl each of mouse anti-human CK-7 monoclonal antibody and goat anti-mouse IgG were added and shaken and incubated at 4°C for 20 min. Flow cytometry was used to detect the CK-7 expression, and the percentage of CK-7 positive expressing cells was used to identify the purity of the cell.

### Cell Transfection

The logarithmic trophoblast cells from normal pregnancy term placenta tissue were taken. When the cells were about 80% of the bottom of the bottle, trypsin digestion and passage and inoculation of 1 × 105 cells per well on a 12-well cell culture plate were performed. At the bottom of the bottle, cells were transfected according to the instructions of the Lipofectamine 2000 kit (Shanghai Duma Biotechnology Co., Ltd.). The Twist1 negative control plasmid and Twist1 interference plasmid were transfected into trophoblast cells, which were the si-NC group and si-Twist1 group, respectively, and the untreated cells were used as the blank control group. Each group has 5 duplicate wells, and the transfection efficiency of the cells was observed 48 h after transfection with a fluorescence microscope.

### Detection of Twist1 Expression

The RT-qPCR and Western blotting were used to detect Twist1 mRNA and protein expression levels in tissues and cells. The TRIzol kit (Shanghai Yihui Biotechnology Technology Co., Ltd.) was used to obtain total RNA, which was subsequently reverse transcribed into cDNA and identified by agarose gel electrophoresis, identification, according to the RT-qPCR SYBR II kit (Shanghai Jizhi Biochemical Technology Co., Ltd.) instructions set the total reaction system to 20 μl, reaction conditions: 94°C pre-denaturation 5 min; denaturation at 95°C for 30 s, annealing at 60°C for 30 s, extension at 72°C for 50 s, repeat 40 cycles; and extension at 72°C for 5 min. Using β-actin as the internal reference gene, the 2^−ΔΔCt^method (12) was used to calculate the expression level of Twist1 mRNA in tissues and cells. Primer sequence: Twist1, Forward: 5′-GAGCTGGACTCCAAGATGG-3′, Reverse: 5′-TTAAGAAATCTAGGTCTCCGGC-3′; β-actin, Forward: 5′-GTGCACACGCACTGCACGCTGCACAC-3′, Reverse: 5′-TCGCCACTCACGTG-3′. The experiment was repeated 3 times and the average value was taken. In Western blotting, the following steps were performed: the tissue or cells were lysed on ice for 30 min, centrifuged and the protein was quantified, the sample was mixed with the loading buffer, denatured in a boiling water bath, centrifuged to collect the supernatant, separated by electrophoresis, wet transferred to the membrane, and sealed with 5% skimmed milk powder at room temperature for 2 h. Then, rabbit anti-rat Twist1 monoclonal antibody (1:1000) was added, incubated overnight at 4°C on a shaker, and goat anti-rabbit IgG secondary antibody (1:8000) was added after washing with TBST and incubated at room temperature for 1 h. It was added to the ECL darkroom for visualization, and the ratio of the gray value of the Twist1 protein band to the gray value of the internal reference β-actin band was used to indicate the protein expression level.

### Detection of Cell Invasion Ability

A Transwell chamber (Shanghai Yanhui Biotechnology Co., Ltd.) experiment was used to detect the cell invasion ability of each group. Matrigel (diluted with PBS to 1 g/L) was pre-laid in a Transwell chamber with 50 μl per well. Cells with a density of 5.0 × 10^5^/ml were added to the upper chamber, and 600 μl containing 10% fetal bovine serum was added to the lower chamber. RPMI medium, routinely cultured for 28 h until the cells degrade Matrigel, took the bottom filter membrane, washed with PBS, fixed with 0.25% glutaraldehyde, stained with 0.1% crystal violet after 20 min, wiped off the upper cells with a sterile cotton swab after 30 min, washed, dried, observed the staining under a microscope. Five fields were randomly selected and the average number of cells passing through the micropores in each field was calculated. This represents the cell invasion ability. The experiment was repeated 3 times and the average value was taken.

### Conception and Grouping of SD Rats

Thirty SD female rats and 60 male rats (Hunan Slike Jingda Laboratory Animal Co., Ltd.) of the same weight and age were selected, and the SD female and male rats were caged together 1:2. The next morning, the vaginal plug was found to be the first day of pregnancy (“vaginal plug,” also known as “vaginal suppository” or “mating suppository,” is a substance formed after the coagulation of semen existing in the vagina after mating of animals for ~24 h [short 4–5 h, long 48 h] to fall off automatically). The pregnant rats were divided into two groups, the normal group and the Twist1 inhibition group, with 8 rats in each group. After the pregnancy was stable, SD rats in different groups were given different treatments. The treatment group was given an intravenous injection of Twist1 antibody, and the control group was given an intravenous injection of normal saline.

### Detection of Mean Tail Artery Pressure

The BP-2000 blood pressure analysis system was used to detect blood pressure before and after the operation. The rat was fixed in a heating bag in the cage, and after preheating at a temperature of 38°C for 10 min, the rat's tail was passed through a pressure sensor and fixed to the root of the rat's tail. The tail artery of the rat was brought into close contact with the pressure sensor, the waveform of the blood pressure measurement system was observed, and the blood pressure was measured after the waveform stabilized. The blood pressure of rats was detected at 1 week, 2 weeks, and full-term; each time, the measurement was repeated 3 times, the systolic and diastolic blood pressure were measured, the average arterial pressure was obtained, where the average arterial pressure = (systolic blood pressure + diastolic blood pressure × 2)/3.

### Detection of Urine Protein Content

The blood pressure was measured and transient urine samples were collected from rats, and urine albumin and creatinine were measured using urine albumin ELISA kit and creatinine detection kit (Shanghai Fantai Biotechnology Co., Ltd.), respectively. The ratio of urine albumin to urine creatinine was used as the criterion for proteinuria. The urinary microalbumin/creatinine ratios of the two groups of rats at 1 week, 2 weeks, and full-term were determined. After the mean tail artery pressure and urine protein were detected at the full term of pregnancy, the pregnancy of SD rats after different treatments was terminated, and the placental tissue was extracted to detect the expression of Twist1 mRNA and protein in rat placental tissue according to the method described in Detection of Twist1 Expression.

### Statistical Methods

The SPSS20.0 statistical software was used for data analysis. All experimental data were normally distributed, and the results were expressed as mean ± *SD*. The comparison between the two groups was performed by an independent sample *t*-test. The difference was statistically significant with *p* < 0.05.

## Results

### The Expression of Twist1 MRNA and Protein in Different Placental Tissues

**Figure 1:**
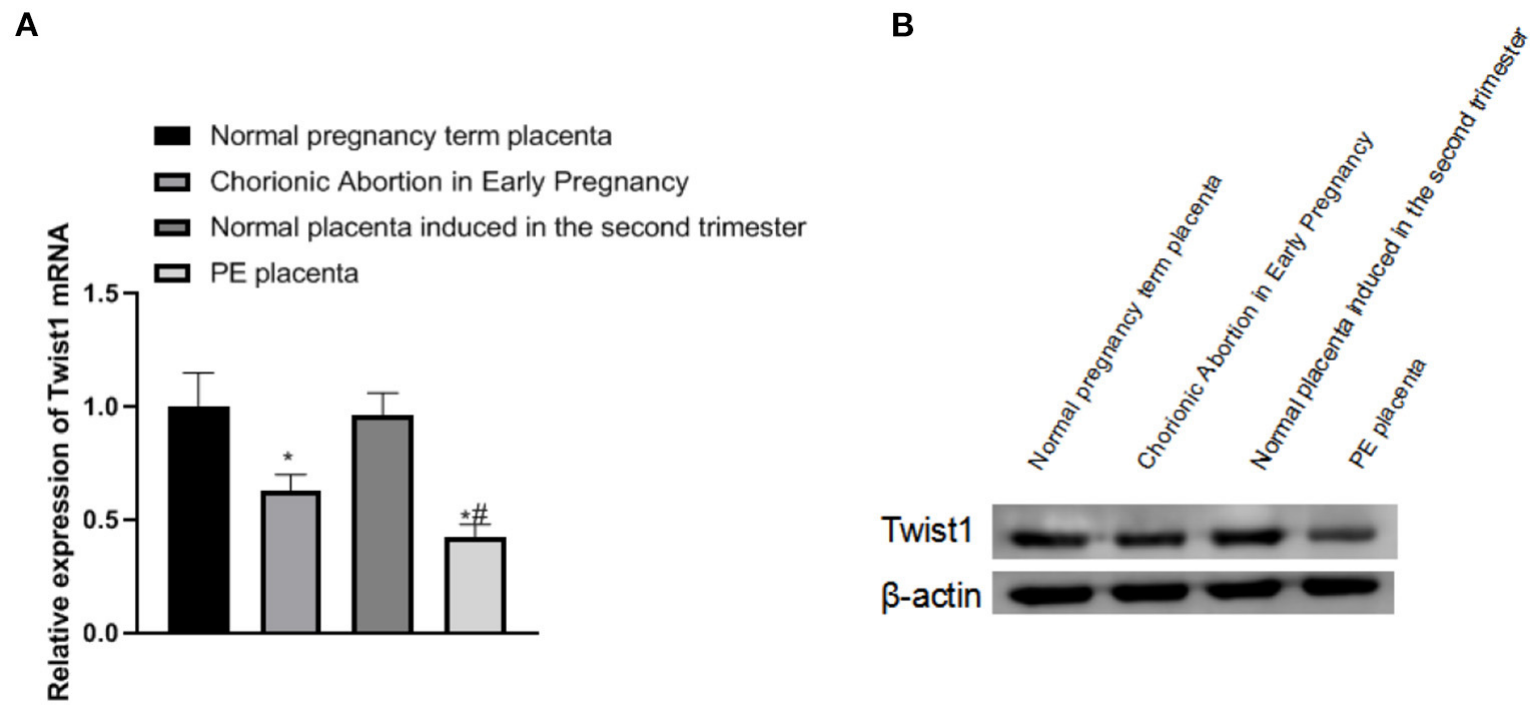
The expression of Twist1 mRNA and protein in different placental tissues. **(A,B)** The expression of Twist1 mRNA and protein; Compared with Normal pregnancy term placenta, *p < 0.05; compared with Chorionic Abortion in Early Pregnancy, ^#^*p* < 0.05.

Compared with normal pregnancy full-term placenta tissue, the expression of Twist1 mRNA and protein in the placenta tissue of patients with early abortion and PE was significantly reduced, and the changes in placenta tissue of patients with PE were more obvious (*p* < 0.05), as shown in Figures 1A,B.

### Identification of Primary Trophoblasts and the Expression of Twist1 MRNA and Protein in the Cells

**Figure 2:**
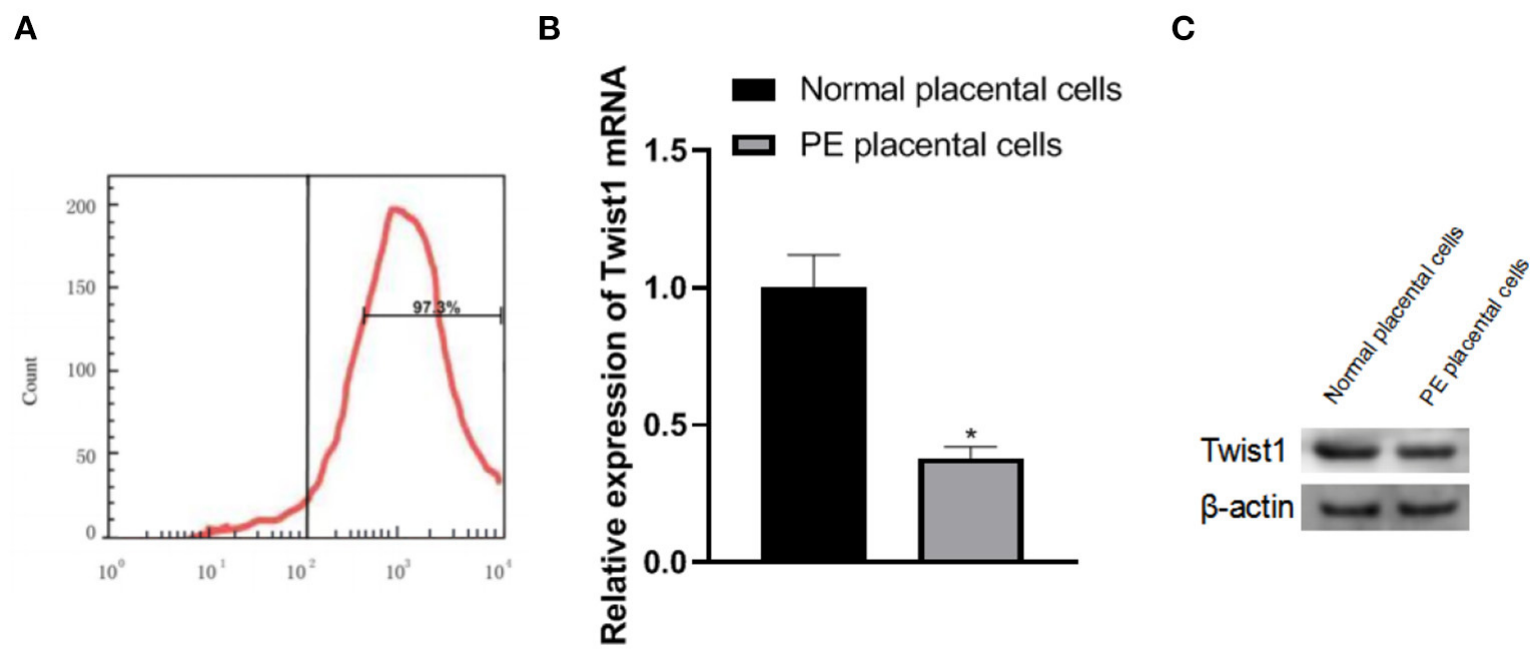
Identification of primary trophoblast cells and the expression of Twist1 mRNA and protein in the cells. **(A)** Identification of Primary Trophoblasts; **(B,C)** The expression of Twist1 mRNA and protein; Compared with Normal placental cells, *p < 0.05.

The primary placental trophoblast cells were detected by flow cytometry. The cell surface-expressed abundant CK-7 and the percentage of CK-7 positive expressing cells were 97.3%, as shown in Figure 2A. The expression of Twist1 mRNA and protein in PE placental cells was significantly lower than that of normal placental cells (*p* < 0.05), as shown in Figures 2B,C.

### The Effect of Interference With Twist1 on Trophoblast Cell Invasion

**Figure 3:**
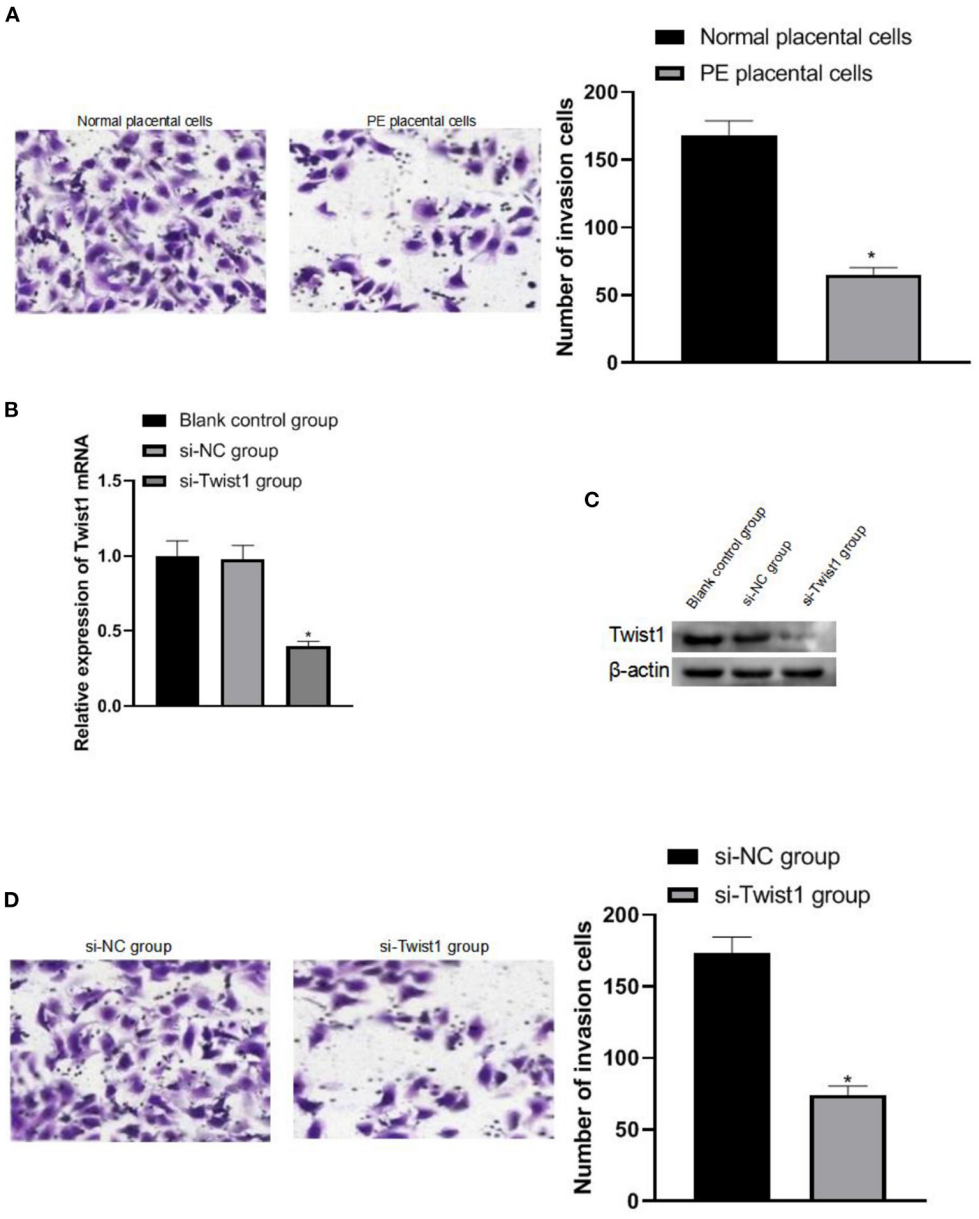
The effect of interference with Twist1 on trophoblast cell invasion. **(A)** Detection of the invasive ability of trophoblasts; **(B,C)** The expression of Twist1 mRNA and protein; **(D)** Detection of the invasive ability of trophoblasts; Compared with Normal placental cells and si-NC group, *p < 0.05.

After the trophoblast cells were isolated, it was observed that the trophoblast cells of PE patients had weaker invasion ability than normal pregnant women's trophoblast cells (*p* < 0.05), as shown in Figure 3A. The invasion ability was significantly weakened (*p* < 0.05), as shown in Figures 3B–D.

### The Effect of Inhibiting Twist1 on Mean Tail Artery Pressure and Urine Protein in Female Mice

**Figure 4:**
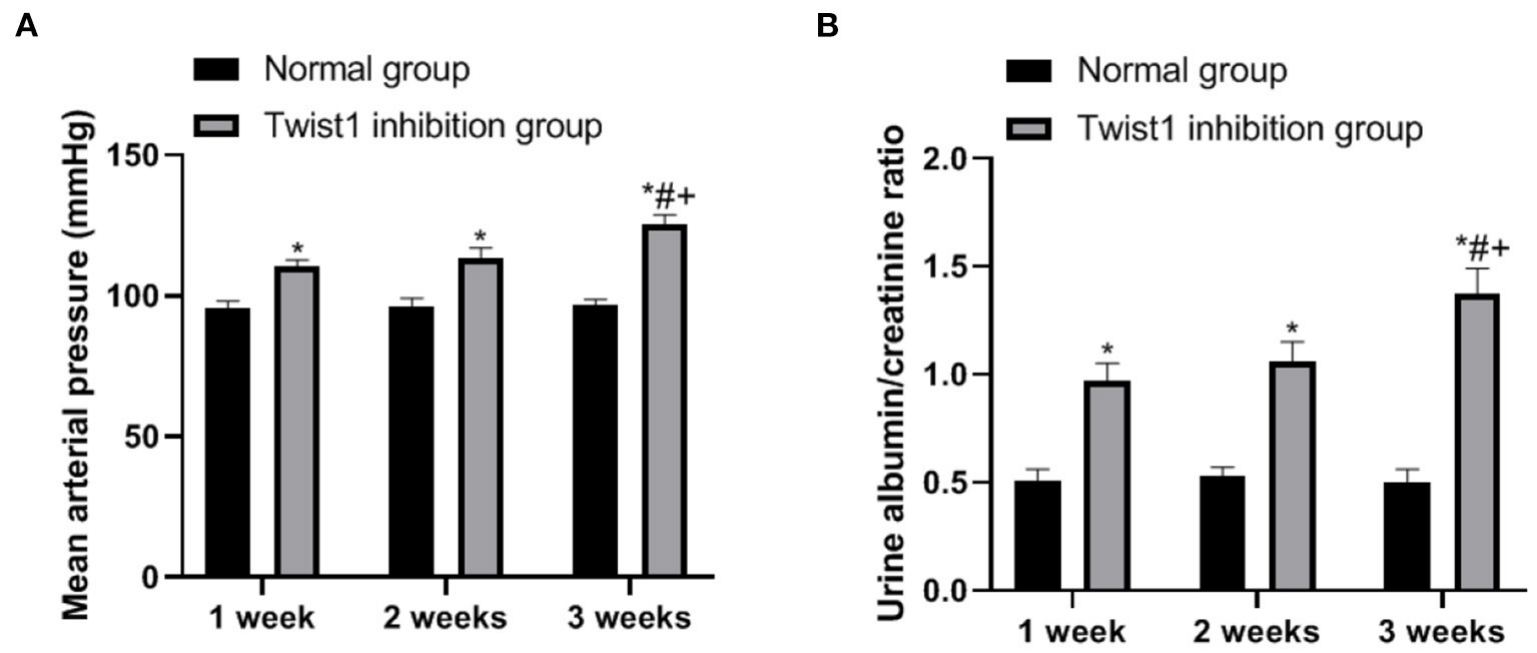
The effect of inhibiting Twist1 on mean tail artery pressure and urine protein in female mice. **(A,B)** Detection of Mean Tail Arterial Pressure and Urine Protein in Female Rats; Compared with Normal group, *p < 0.05; compared with Twist1 inhibition group of 1 week, ^#^*p* < 0.05; compared with Twist1 inhibition group of 2 week, ^+^*p* < 0.05.

The observation of *SD* conceived rats with different treatments found that compared with the normal group, the average tail artery pressure and urine protein level of rats in the Twist1 inhibition group increased significantly at 1, 2, and 3 weeks (*p* < 0.05), as shown in Figures 4A,B.

### The Expression of Twist1 MRNA and Protein in the Placenta of Two Pregnant Rats

**Figure 5:**
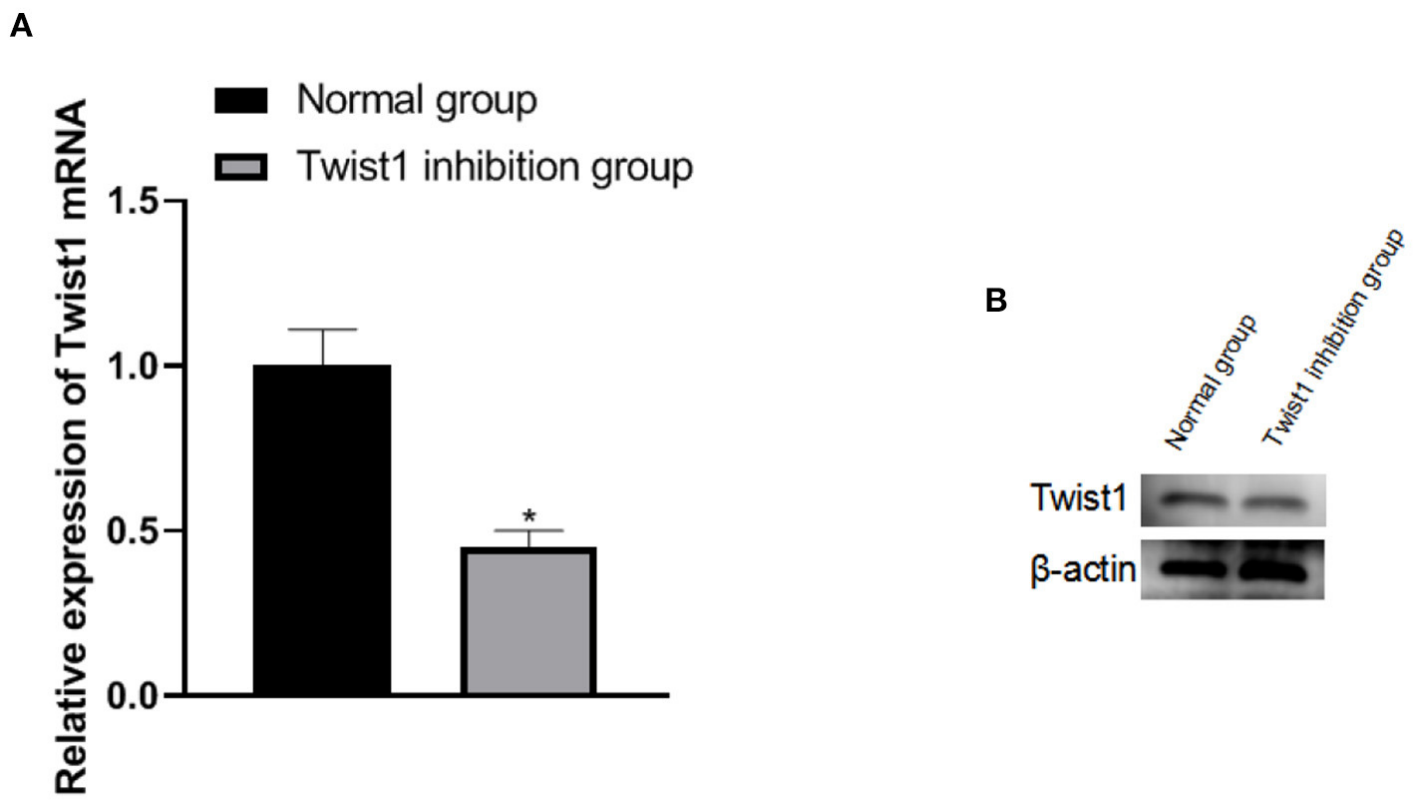
The expression of Twist1 mRNA and protein in the placenta of two pregnant rats. **(A,B)** The expression of Twist1 mRNA and protein; Compared with Normal group, *p < 0.05.

After the term, the expression of Twist1 in the placenta tissue was detected and it was found that the expression of Twist1 mRNA and protein in the placenta tissue of the Twist1 inhibition group was significantly lower than that of the normal group (*p* < 0.05), as shown in Figures 5A,B.

## Discussion

Preeclampsia manifests as hypertension, proteinuria, and is often accompanied by edema. PE with severe clinical manifestations is called severe PE, including severe hypertension, abnormal nerve, and circulatory and organ dysfunction (13). If PE is not well controlled, it can progress to eclampsia. It is possible to develop a variety of complications that threaten the lives of mothers and children, including hemolysis, elevated creatinine levels, low platelet syndrome, placental abruption, multiple organ dysfunction, diffuse intravascular coagulation, fetal distress, fetal intrauterine growth restriction, and stillbirth (14–16). Worldwide, the incidence of PE fluctuates from 5 to 12%, and the mortality rate of patients with eclampsia can reach 1%. Epidemiological investigations show that high-risk factors for PE include advanced age (≥40 years), diabetes, obesity, multiple pregnancies, family history of PE, and thrombotic vascular disease. The etiology and pathogenesis of PE have not yet been fully elucidated. At present, it is generally believed that the occurrence and development of PE involve a variety of factors, such as abnormal trophoblast cell invasion, abnormal immune regulation, endothelial cell damage, genetic factors, and systemic inflammation (17–19).

Trophoblasts are the main component of the placental structure and play an important role in the formation of the placenta and the maintenance of the normal development of the fetus. In the early stage of blastocyst implantation, trophectoderm cells differentiate into two types of trophoblast cells, namely cytotrophoblast cells and syncytial trophoblast cells (20). In the early stage of blastocyst implantation, trophectoderm cells differentiate into two types of trophoblast cells, namely cytotrophoblast cells and syncytial trophoblast cells. There are two different differentiation pathways for cytotrophoblast cells: One is to fuse with syncytiotrophoblast cells, and the other one is to differentiate into infiltrating extravillous trophoblast cells (EVTs). Some EVTs infiltrate the deep layer of the endometrium to the inner third of the myometrium and are called interstitial trophoblast cells; some of the trophoblast cells that invade the maternal uterine spiral artery are called intravascular trophoblast cells. There are two stages of EVTs, invasion and placenta formation during pregnancy. In the first stage, 10 weeks before pregnancy, EVTs successfully invaded the decidua tissue of the uterus. If this process is blocked, it will lead to miscarriage. In the second stage, 10–18 weeks, EVTs successfully invade the subdecidual muscle layer and uterine SPA. The endothelial cells and smooth muscle cells of SPA undergo apoptosis, and gradually replace the vascular endothelium and vascular smooth muscle cells (VSMCs). The vascular basement membrane is degraded. The elastic fibers of the tube wall degeneration to form fibrin-like substances, which lead to changes in vascular resistance, forming a high-flow, low-resistance SPA, ensuring that sufficient circulating blood flow is provided for the fetal placenta (21). Therefore, damage to trophoblasts may also lead to the development of PE.

In female reproductive function, Twist1 can regulate the decidualization of uterine stromal cells, embryonic development, and organ differentiation (22). The development of the placenta and tumor growth has very similar characteristics in many aspects, so it can be speculated that the regulatory mechanism of Twist1 on tumor cell apoptosis also acts on trophoblast cells (23). In the study, focusing on the changes and nourish PE contact cell activity and function, by regulating the expression Twist1 detected trophoblast cell invasion ability change, thereby further exploring the role of Twist1 in placental development and pathogenesis of PE. The study found that compared with normal pregnancy full-term placenta tissue, the expression of Twist1 mRNA and protein in the chorionic tissue of early abortion and the placenta of PE patients were significantly reduced, and the chorionic tissue of the placental tissue of PE patients was significantly reduced compared with that of the early stage aborted chorionic tissue, which is partly consistent with the research results of Ma et al. (10). Twist1 is an evolutionarily highly conserved transcription factor, which is widely expressed in a variety of normal tissues and tumor cells and plays a regulatory role (24). Due to insufficient gestational age in abortion patients, the placental tissue of PE patients presents pathological changes. The decrease of Twist1 expression may indicate that it is related to the gestational age and pathological changes.

In order to explore the role of Twist1 in PE, linked it with trophoblasts, after we separated the trophoblasts, we observed that the trophoblasts of PE patients were weaker than the trophoblasts of normal pregnant women, suggesting that impaired trophoblast invasion may be the cause of the development of PE. After interfering with Twist1, it was found that the invasion ability of cells was also significantly weakened, suggesting that Twist1 can regulate the invasion ability of trophoblasts.

This also explains the reason for the low expression of Twist1 in the placental tissues of PE patients. The reduced expression of Twist1 weakens the invasion ability of trophoblasts, hinders the invasion of trophoblasts, and leads to obstacles to SPA remodeling. At the same time, oxygen free radicals are released and the vascular endothelium is damaged, causing the production of PE. Animal experiments were conducted to explore the effects of Twist1 on the body. Observation of SD conceived rats with different treatments found that after Twist1 inhibition, the average tail artery pressure and urine protein levels of the rats increased significantly at 1 week, 2 weeks, and full-term. Hypertension and proteinuria are the main manifestations of PE. After the term, the expression of Twist1 in the placenta tissue was detected, and it was found that the expression of Twist1 mRNA and protein in the placenta tissue of the Twist1 inhibition group was significantly lower than that of the normal group. It shows that the *in vivo* experiment interferes with the success of Twist1, and the insufficient expression of Twist1 can make pregnant female mice show PE morbidity.

## Conclusion

In summary, the expression of Twist1 is downregulated in PE, and the invasion ability of trophoblast cells in the placental tissue of PE patients is weak. After inhibiting Twist1, the mean tail artery pressure and urine protein level of SD conceived rats increased, showing a trend of PE. The mechanism may be related to the ability of Twist1 to inhibit the invasion of placental trophoblast cells.

## Data Availability Statement

The original contributions presented in the study are included in the article/Supplementary Material, further inquiries can be directed to the corresponding author.

## Ethics Statement

The studies involving human participants were reviewed and approved by this study was approved by the Ethics Committee of the First Affiliated Hospital, Hengyang Medical School, University of South China. The patients/participants provided their written informed consent to participate in this study. The animal study was reviewed and approved by this study was approved by the Animals Ethics Committee of the First Affiliated Hospital, Hengyang Medical School, University of South China.

## Author Contributions

WT is responsible for the design of the study and the conduct of the experiment. SY is responsible for the detection of the results, the statistics of the data and the writing of the article, and YL is the supervisor of the whole study. All authors of this study have made equal contributions. All authors contributed to the article and approved the submitted version.

## Funding

This study was supported by the 2021 Hunan Provincial Health Commission Scientific Research Project (202105020156).

## Conflict of Interest

The authors declare that the research was conducted in the absence of any commercial or financial relationships that could be construed as a potential conflict of interest.

## Publisher's Note

All claims expressed in this article are solely those of the authors and do not necessarily represent those of their affiliated organizations, or those of the publisher, the editors and the reviewers. Any product that may be evaluated in this article, or claim that may be made by its manufacturer, is not guaranteed or endorsed by the publisher.
