# Supplementary figures and images for: The Mechanism of Downregulation of Twist1 Inhibiting Trophoblast Invasion and Aggravating the Development of Preeclampsia
Source: Front Surg. 2022 Mar 17;9:862716. doi: 10.3389/fsurg.2022.862716 (PMC8968441; doi:10.3389/fsurg.2022.862716)

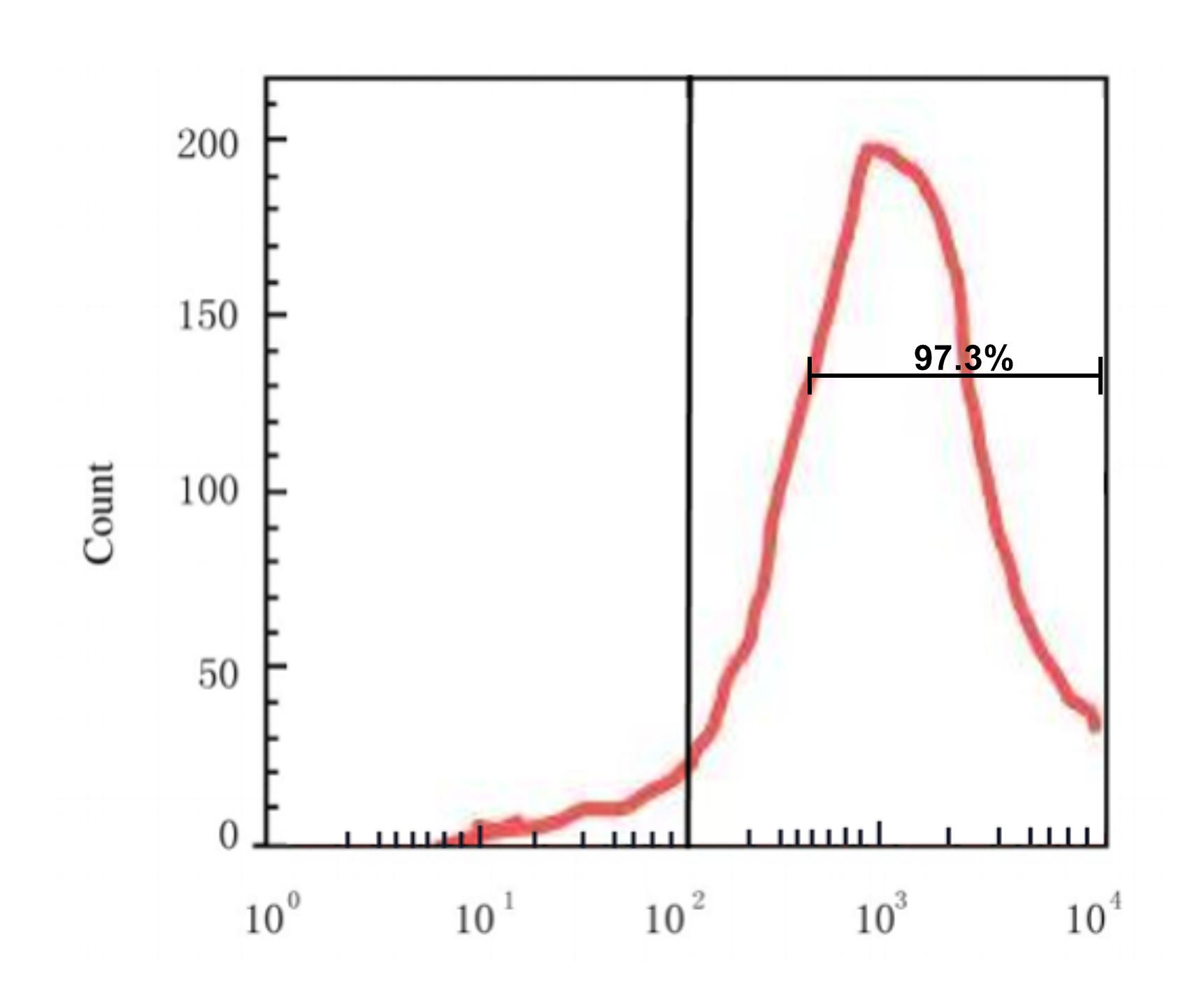

Supplement: Supplementary file 1 [file Data_Sheet_1.ZIP › Source Data/Original Picture/Flow Cytometry.jpg]

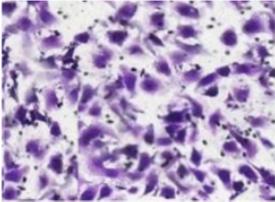

Supplement: Supplementary file 1 [file Data_Sheet_1.ZIP › Source Data/Original Picture/Transwell/1.jpg]

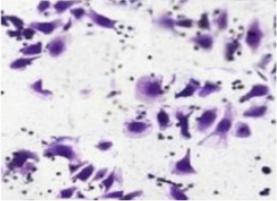

Supplement: Supplementary file 1 [file Data_Sheet_1.ZIP › Source Data/Original Picture/Transwell/2.jpg]

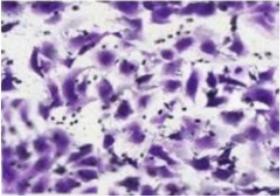

Supplement: Supplementary file 1 [file Data_Sheet_1.ZIP › Source Data/Original Picture/Transwell/3.jpg]

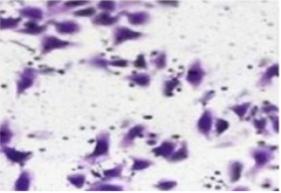

Supplement: Supplementary file 1 [file Data_Sheet_1.ZIP › Source Data/Original Picture/Transwell/4.jpg]

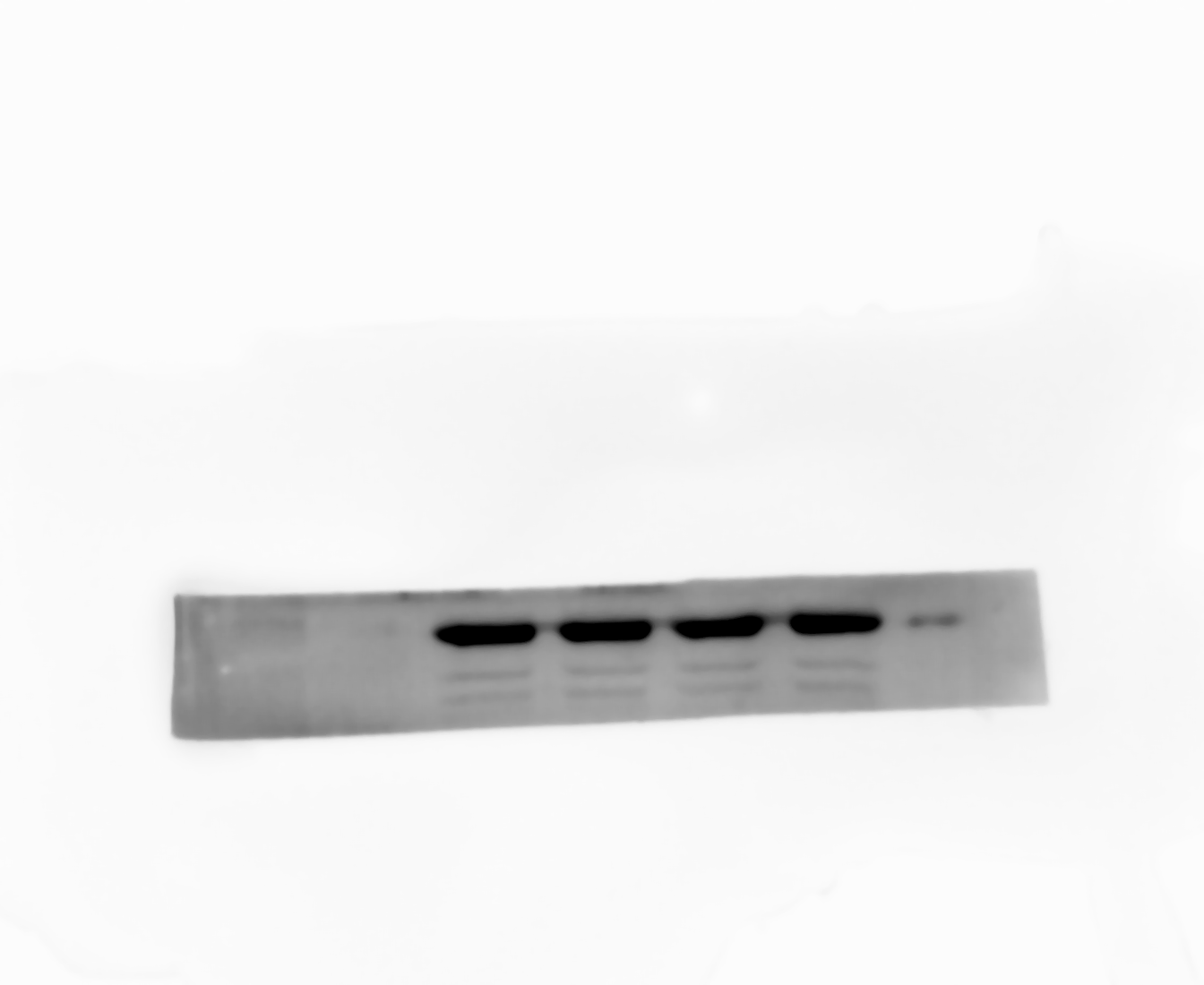

Supplement: Supplementary file 1 [file Data_Sheet_1.ZIP › Source Data/Original Picture/Western blotting/B-actin 1 .tif]

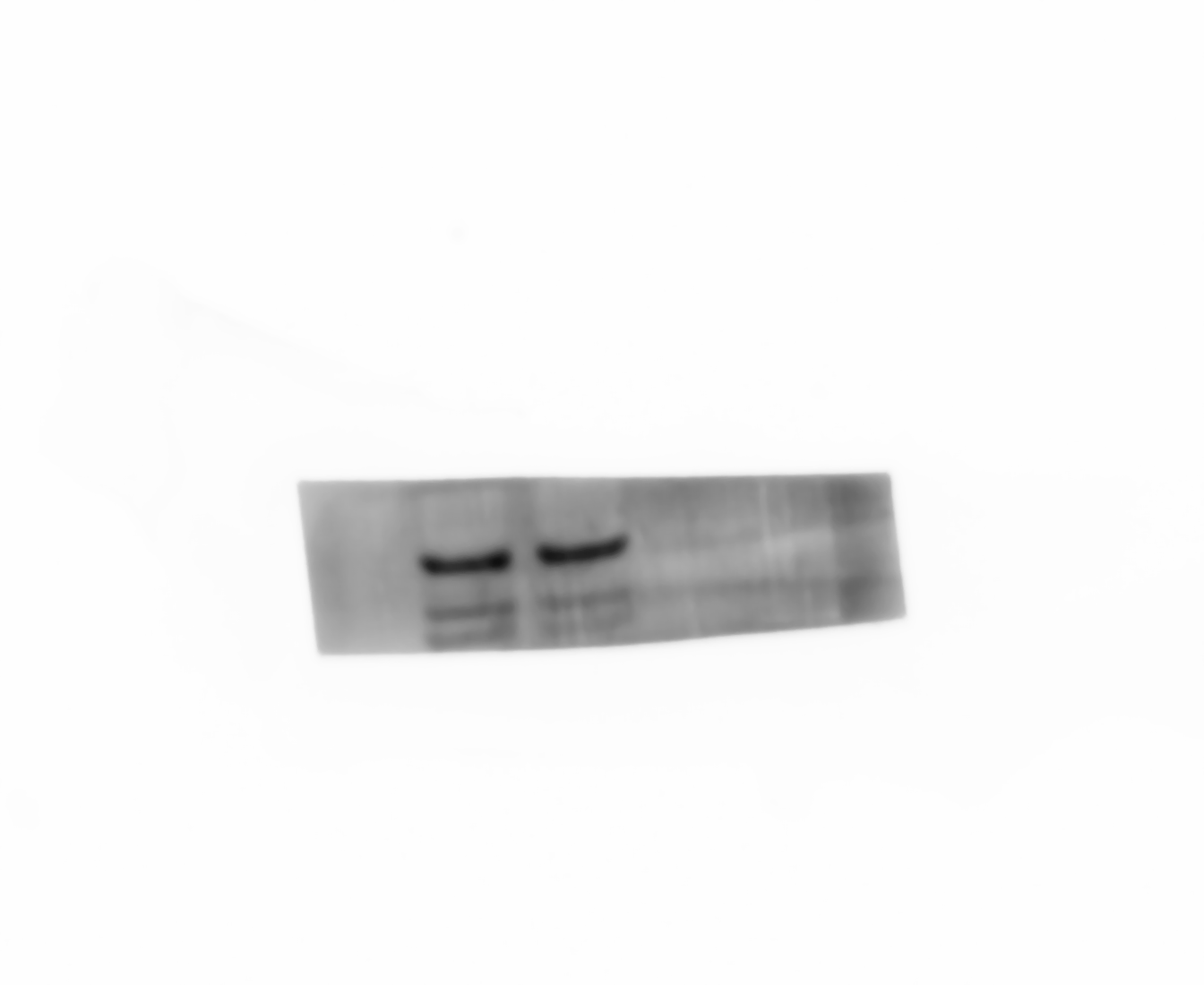

Supplement: Supplementary file 1 [file Data_Sheet_1.ZIP › Source Data/Original Picture/Western blotting/B-actin 2.tif]

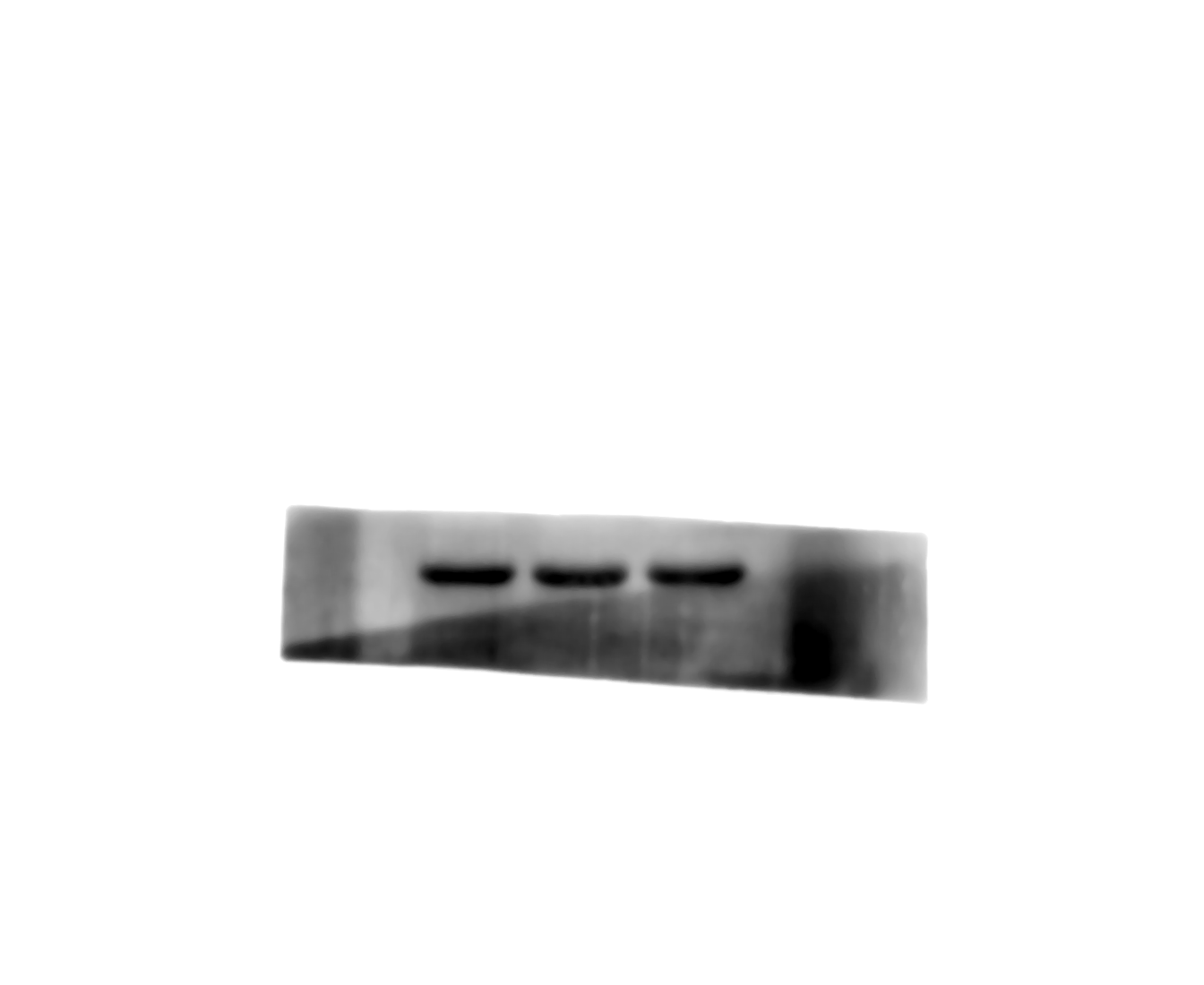

Supplement: Supplementary file 1 [file Data_Sheet_1.ZIP › Source Data/Original Picture/Western blotting/B-actin 3.tif]

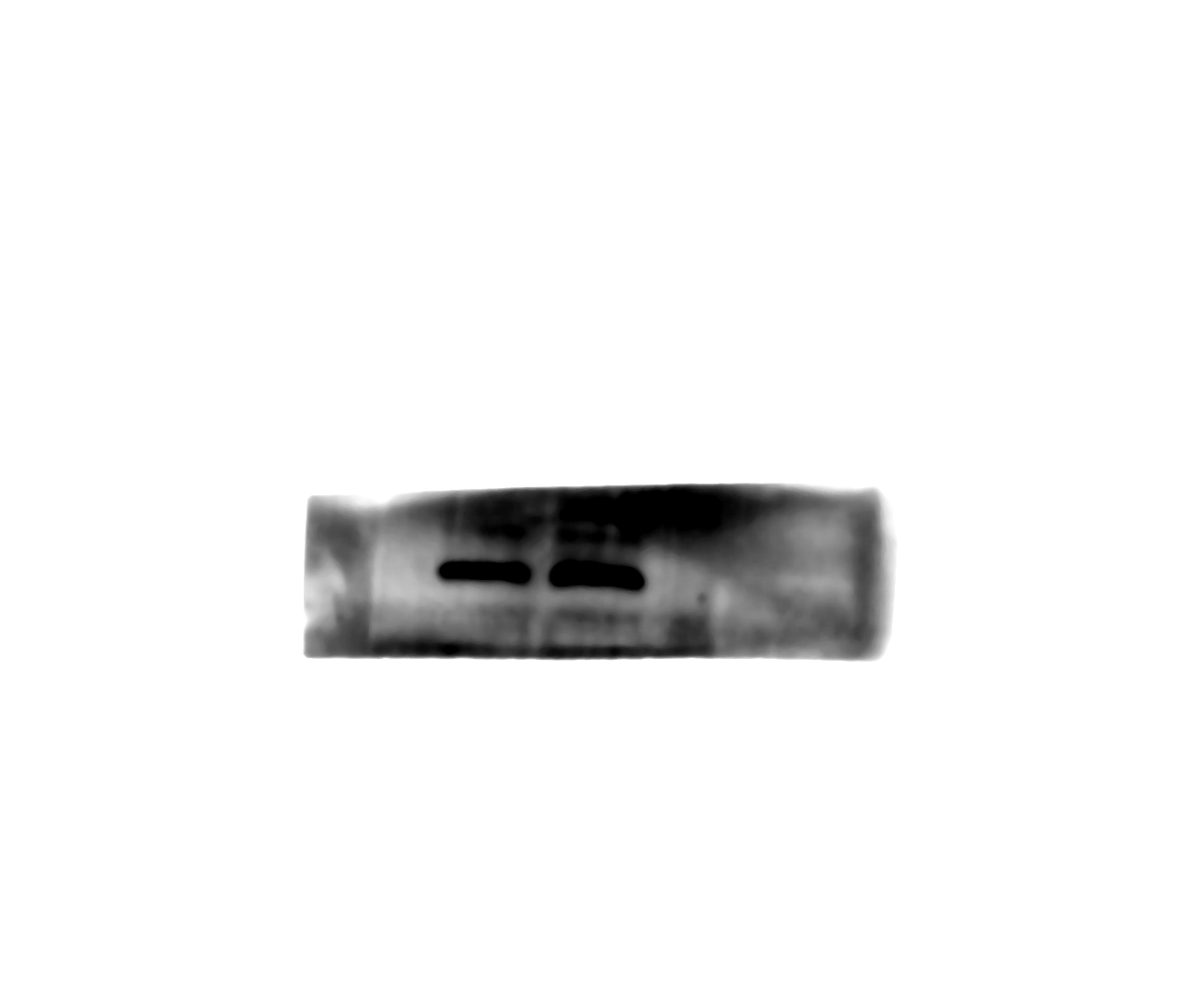

Supplement: Supplementary file 1 [file Data_Sheet_1.ZIP › Source Data/Original Picture/Western blotting/B-actin 4.tif]

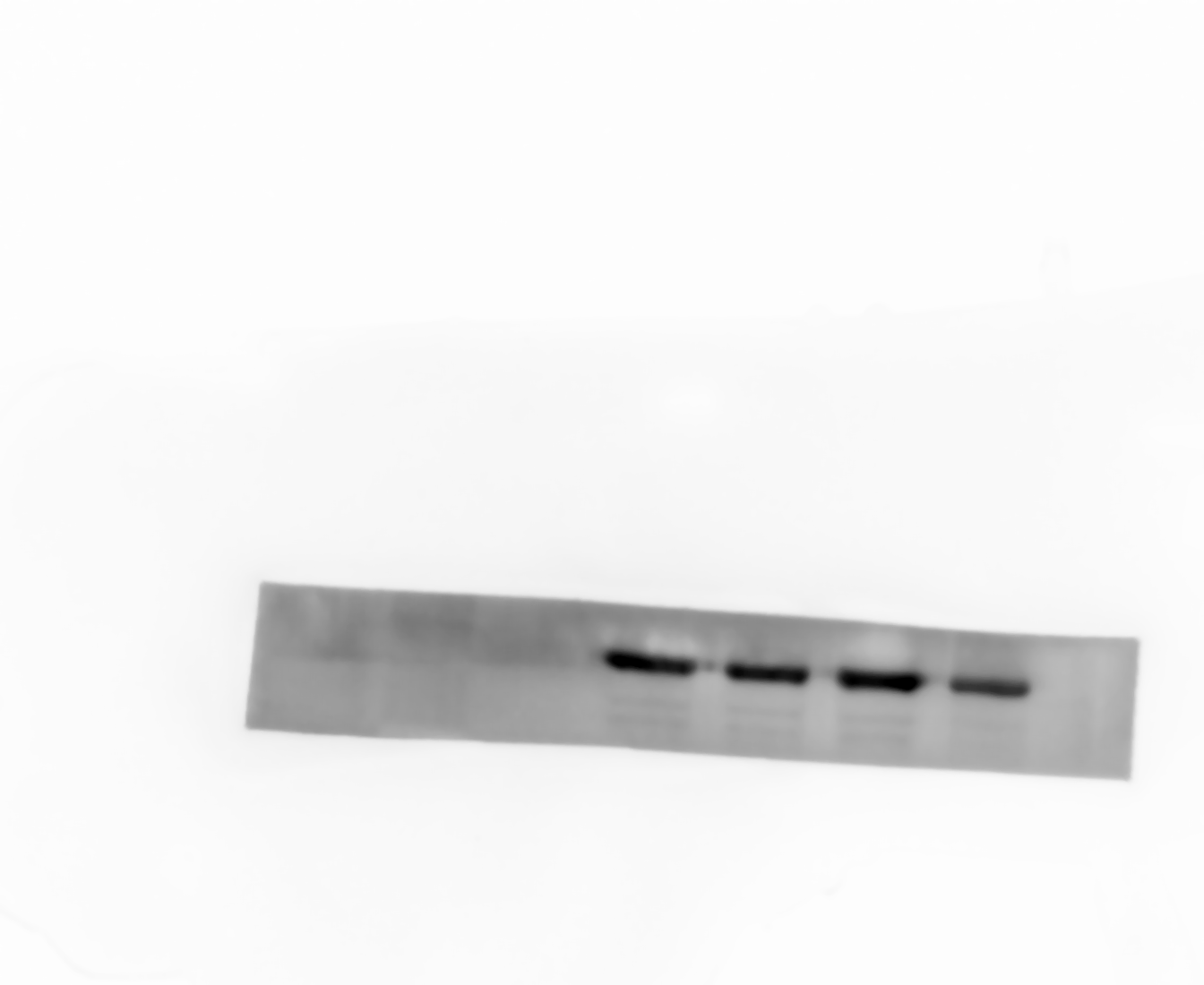

Supplement: Supplementary file 1 [file Data_Sheet_1.ZIP › Source Data/Original Picture/Western blotting/twist1 1.tif]

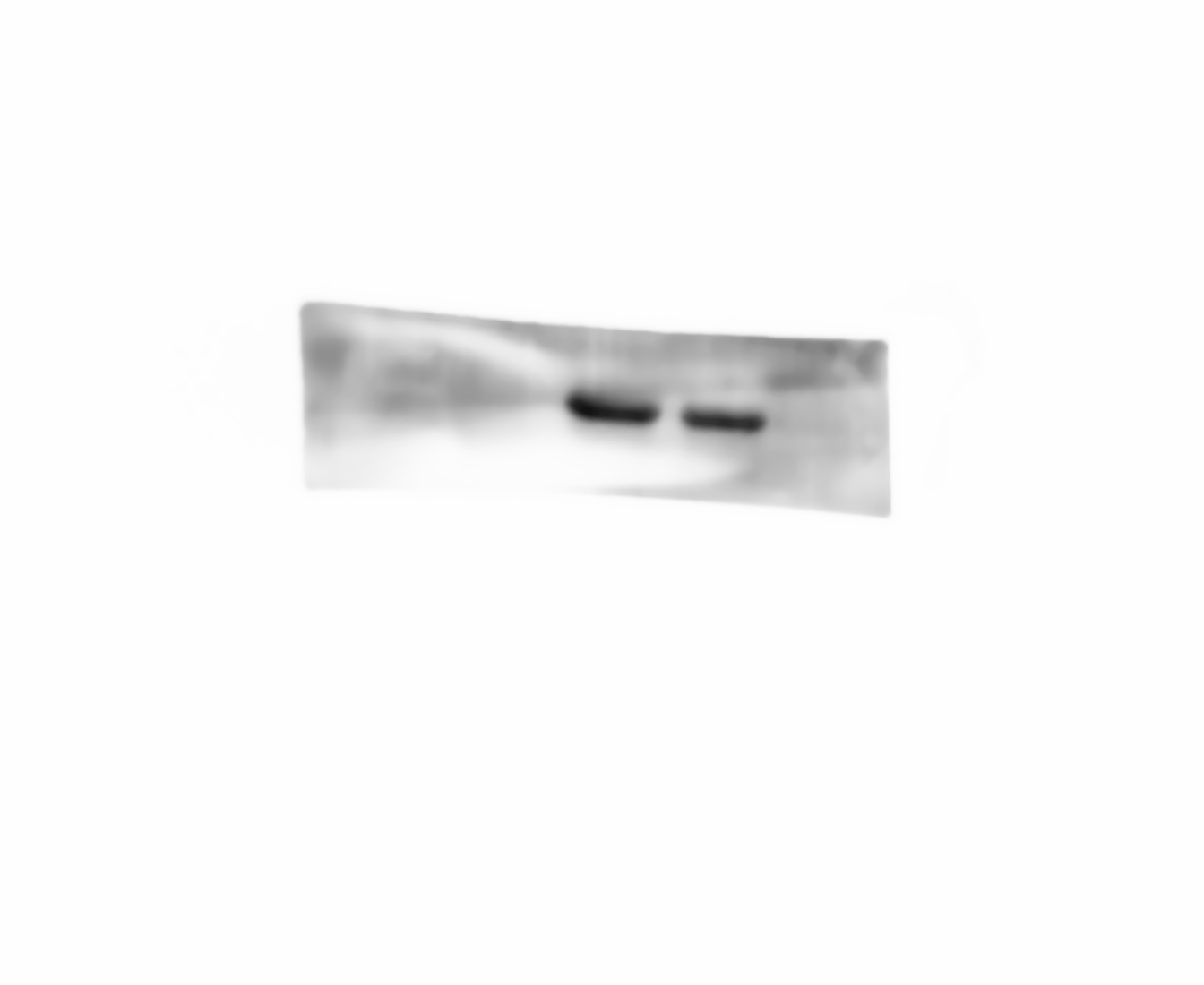

Supplement: Supplementary file 1 [file Data_Sheet_1.ZIP › Source Data/Original Picture/Western blotting/Twist1 2.tif]

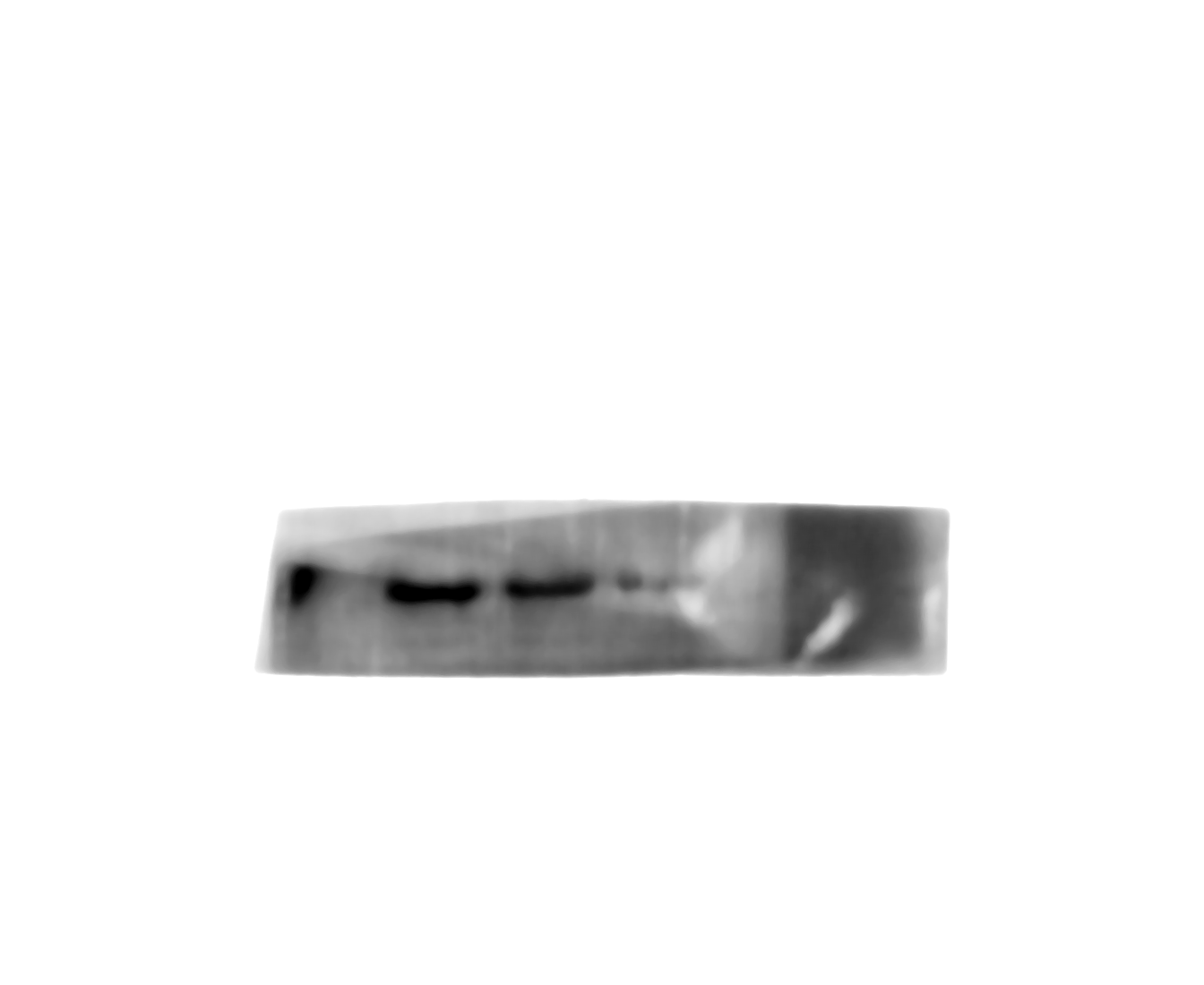

Supplement: Supplementary file 1 [file Data_Sheet_1.ZIP › Source Data/Original Picture/Western blotting/twist1 3.tif]

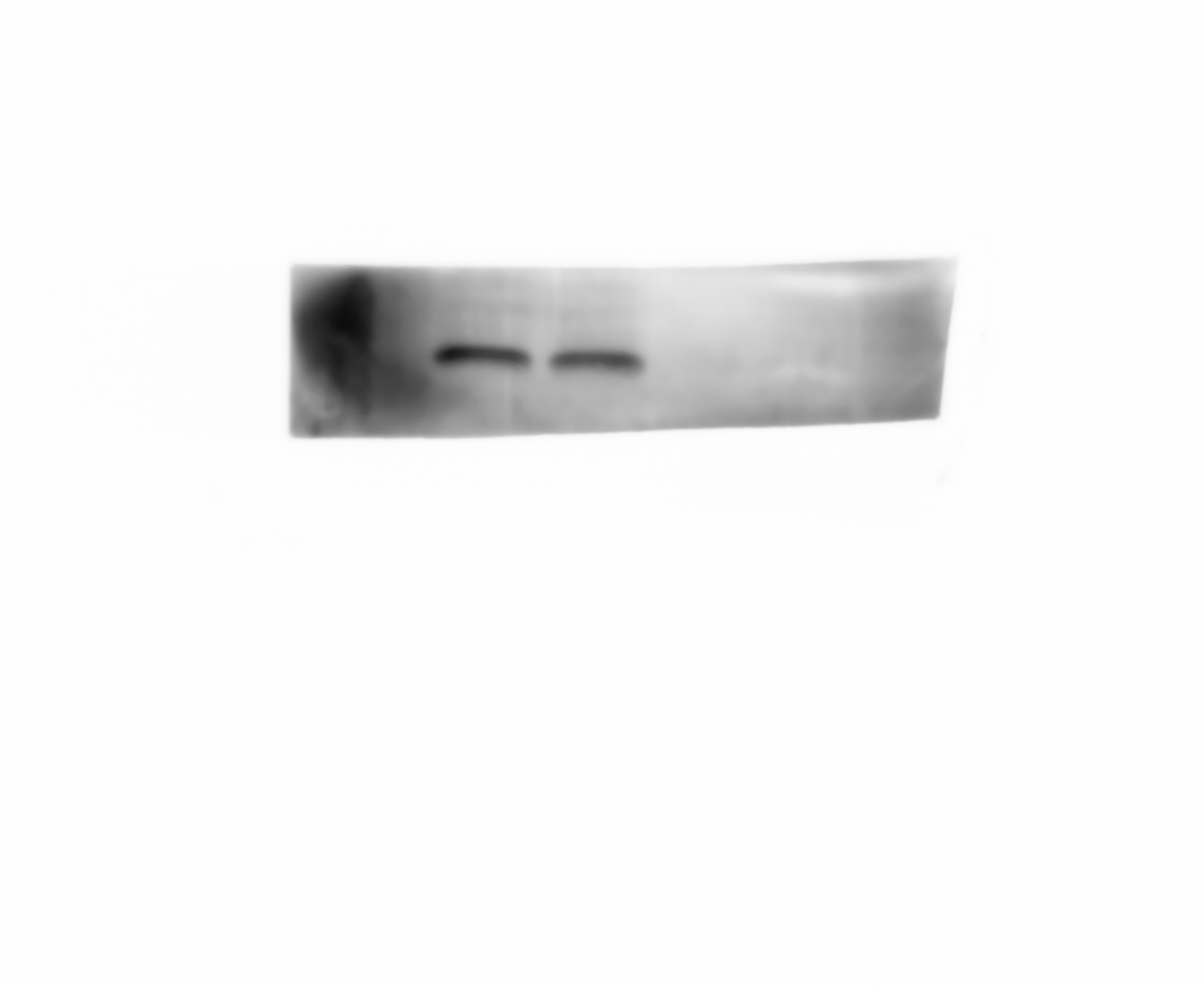

Supplement: Supplementary file 1 [file Data_Sheet_1.ZIP › Source Data/Original Picture/Western blotting/Twist1 4.tif]

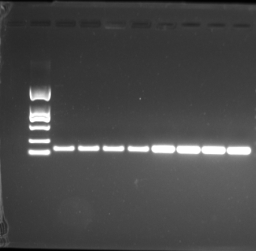

Supplement: Supplementary file 1 [file Data_Sheet_1.ZIP › Source Data/Electropherogram/qPCR result electropherogram/1.png]

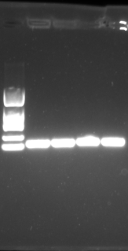

Supplement: Supplementary file 1 [file Data_Sheet_1.ZIP › Source Data/Electropherogram/qPCR result electropherogram/2.png]

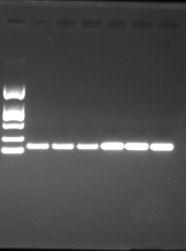

Supplement: Supplementary file 1 [file Data_Sheet_1.ZIP › Source Data/Electropherogram/qPCR result electropherogram/3.png]

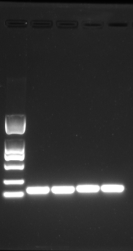

Supplement: Supplementary file 1 [file Data_Sheet_1.ZIP › Source Data/Electropherogram/qPCR result electropherogram/4.png]

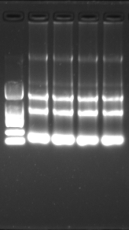

Supplement: Supplementary file 1 [file Data_Sheet_1.ZIP › Source Data/Electropherogram/RNA electropherogram/1.png]

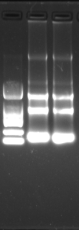

Supplement: Supplementary file 1 [file Data_Sheet_1.ZIP › Source Data/Electropherogram/RNA electropherogram/2.png]

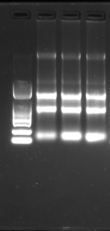

Supplement: Supplementary file 1 [file Data_Sheet_1.ZIP › Source Data/Electropherogram/RNA electropherogram/3.png]

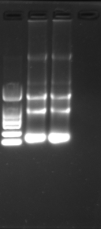

Supplement: Supplementary file 1 [file Data_Sheet_1.ZIP › Source Data/Electropherogram/RNA electropherogram/4.png]
